# Content validation of the COST for patient questionnaire (COPAQ) for patients with low back pain: a qualitative study

**DOI:** 10.1017/S0266462324000515

**Published:** 2024-11-05

**Authors:** Layla Bakaa, Fatima Al-Mosawi, Nora Bakaa, Lisandra Almeida de Oliveira, Maude Laberge, Luciana G. Macedo

**Affiliations:** 1 Department of Epidemiology, Harvard T.H. Chan School of Public Health, Boston, MA, USA; 2 School of Rehabilitation Science, McMaster University, Hamilton, ON, Canada; 3 Operations and Decision Systems Department, Faculty of Administrative Sciences, Laval University, Quebec City, QC, Canada

**Keywords:** low back pain, cost for patients questionnaire, direct costs, health care utilization, content validity

## Abstract

**Introduction:**

The costs of low back pain (LBP) are complex and difficult to estimate. This study aims to adapt the Cost for Patients Questionnaire (CoPaQ) for use in LBP populations.

**Materials and methods:**

In a cross-sectional qualitative study, we conducted cognitive interviews to assess the CoPaQ’s suitability for addressing costs related to LBP. Three groups of participants were included (*n* = 5 each): (i) persons with a history of LBP or primary caregiver, (ii) researchers with expertise in LBP, and (iii) primary care providers specialized in treating LBP. The interpretation, analysis, and summary of results used Knafl et al.’s qualitative content analysis method.

**Results:**

Persons with a history of LBP (*n* = 5), had a median age of 60 years (Interquartile Range (IQR): 26–71.5), and varying durations of LBP, the median duration of LBP 7 years (IQR: 4–32.5). Researchers (*n* = 5) had a median age of 33 years (IQR: 29–45). Primary care providers (*n* = 5) had a median age of 40 years (IQR: 37.5–65), and a background in chiropractic care (*n* = 3) and physiotherapy (*n* = 2). Content analysis of the interviews revealed sources of error with five pre-determined themes (clarity/comprehension, relevance, inadequate response definition, reference point, perspective modifiers) and one developed theme (organization). We modified the questionnaire for LBP populations based on the feedback.

**Conclusion:**

Our study evaluated the content validity of a questionnaire that assesses the direct and indirect costs associated with LBP. Future studies should pilot this questionnaire with persons of varying LBP severity and compare it with cost diaries.

## Introduction

Low back pain (LBP) is the largest contributor to years lived with disability worldwide (1) and affects several life domains (2) including limitation in daily activities such as work, housework, and leisure activities. LBP affects relationships, increases feelings of loneliness, depression, cohabitation problems, and social participation (3). The annual cost of medical expenditures for LBP in Canada is estimated to be between $6 and $12 billion (4), excluding the impact on society such as loss in work productivity and workers’ disability (5). Direct healthcare costs are those incurred by the patient in their utilization of services or treatment (6-8). Indirect costs are recognized as the highest cost factor for LBP and reflect opportunity costs related to secondary consequences of LBP, that is, losses resulting from work absenteeism and informal caregiving (9;10). Direct healthcare costs, such as medical specialist care, hospital costs, and rehabilitation for LBP, and indirect costs in the United States are estimated to exceed $100 billion (8;11-13). Estimates of costs vary greatly depending on study methodology; however, the estimates overall suggest that LBP in Canada and the United States are a substantial burden on society and the individual.

Economic evaluations of interventions typically focus on costs to the healthcare system (hospitals or the health insurer (public or private)) (14) but fail to consider costs to patients and the community. Cost diaries have often been used to estimate these expenses (15); however, cost diaries are labor-intensive, thus more costly, and require more motivation from patients and researchers to complete. Thus, we propose to use a questionnaire assessing the costs of LBP more holistically, using information typically collected in a diary.

The Costs for Patients Questionnaire (CoPaQ) is a questionnaire developed for an ambulatory population and has several stages of validation (16). Researchers conducted a Delphi study with a panel of experts including health economists, clinicians, and patients and through four rounds, identified specific elements of the questionnaire and refined the wording. A communication specialist was also involved to ensure that the language would be understandable for the lay public (16). The CoPaQ is available in French and English and its temporal stability was assessed using a test–retest design. This questionnaire consists of three parts. The first section has six questions (a total of thirty-one sub-questions) related to costs such as: costs patients need to cover, average time spent (or required) to access medical services, costs related to patient’s job, financial stress caused by patient’s state of health, costs for the caregiver, time spent by patient’s caregiver. The second section contains sociodemographic information and other individual characteristics. There is a third section with a free-text box for respondents to add any additional information that they want. This questionnaire was developed for a general chronic disease and has yet to be validated in a chronic pain population.

Given that there are no validated questionnaires or tools to estimate the costs of LBP, we propose to adapt the CoPaQ for use in this patient population and assess the content validity of the questionnaire. Content validity is the first step in the development of a questionnaire and describes the degree to which items of an assessment tool are representative of the entire domain the assessment tool seeks to measure (17). According to the consensus-based standards for the selection of health measurement instruments (COSMIN) guidelines, content validity is best assessed using qualitative research methods (18). Thus, the primary aim of this study was to evaluate the content validity of the questionnaire through cognitive interviews with persons with a history of LBP or primary caregivers, researchers with expertise in LBP, and primary caregivers specialized in treating LBP (e.g., physiotherapists, chiropractors). We reported the cognitive interview diagnosis for coherence measurement cases. The final goal of this study was to adapt the questionnaire to LBP patients, based on the cognitive interview results, which included re-wording questions to the LBP context or the identification of any potential items that may not have been included in the CoPaQ.

## Methods

### Study design

This is a cross-sectional qualitative study that aims to inform and assess the CoPaQ for use in the context of LBP. Content validity refers to the extent to which the content of an instrument represents the construct it intends to measure (17). According to COSMIN guidelines, ensuring content validity requires establishing relevance, comprehensiveness, and comprehensibility of the instrument, emphasizing that this form of validation necessitates a qualitative approach (18). We used qualitative cognitive interview data collection and analytical techniques, specifically the Think-Aloud (TA) and Verbal Probing (VP) methods (19-21). Participants were invited to express their thought processes and provide feedback on specific elements of the questionnaire. These techniques capture the participants’ reasoning and understanding, which helps ensure that the questionnaire captures the concept it intends to measure, and helps to identify potential problems in the questionnaire before it is widely used, ensuring content validity and acceptability (19;20;22;23). When completing the questionnaire using the TA method, participants were not given a timeframe for the completion of the questionnaire (e.g., costs over the last 3 months), allowing for a more in-depth engagement. We received ethics approval from the Hamilton Integrated Research Ethics Board (#13872).

### Sampling and participants

We used a purposeful sampling technique to select fifteen information-rich participants (participants with lived experience of specific expertise related to LBP) (15;24). The participants comprised three groups: (i) primary care providers (e.g., physicians, physiotherapists, chiropractors) working in Ontario with a specialization in managing LBP; (ii) LBP researchers (e.g., professors or graduate students in Health Economics, Health Management, and Rehabilitation Science); and (iii) persons with a history of LBP or primary caregiver. Researchers were selected to provide insight into the metrics necessary to collect in calculating costs, patients were informed of the day-to-day costs associated with managing and treating LBP, as well as indirect costs, and primary care providers can help narrow down costs based on their experience of treatment or inform missing items.

All participants were required to speak and read English and be over the age of 18. The persons with LBP were required to meet the following criteria:

- chronic non-specific LBP (>3 months) with/without leg pain,
- back pain is the primary musculoskeletal complaint of the patient,
- or be an informal caregiver to a person meeting the abovementioned criteria.

Cognitive interviews took place one-on-one with each participant via Zoom that also was used to generate preliminary transcripts.

### Recruitment

We recruited fifteen participants. Primary care providers and LBP researchers were contacted through the research team network or collaborators through email until the minimum number of participants per group was recruited or until the saturation of themes was met. Potentially eligible patients were approached by their treating primary care provider, who collaborated with our research on multiple projects. Patients were also recruited through an email invitation sent to a list of patient partners and were provided with the contact information of the research team. No primary caregivers were identified.

All participants received an electronic copy of the questionnaire and informed consent form prior to the interview date via email. Participants’ demographic information, or professional backgrounds (if applicable) were collected. Verbal informed consent was obtained during the interviews. Data were de-identified using a coding system to maintain the anonymity of the participants when using illustrative quotes.

### Cognitive interviewing procedure

LB conducted approximately 30-minute interviews via Zoom with NB or LA. LB was an undergraduate student with 2 years of work experience in public health and 1 year of experience with conducting qualitative research. NB and LA are health care professionals and researchers working with this patient population and with qualitative research. The interviewer started by explaining the purpose of the interview, and the interview process, which consisted of two parts. In the first part, the interviewer used the TA technique while going through the CoPaQ with the participant. In the second part, the interviewer used the VP technique to further elicit participants’ thoughts on the questionnaire, which consisted of a semi-structured interview using the interview guide developed for this study (Appendix A of the Supplementary Material). Verbal consent for the qualitative interviews was taken by the interviewer at the time of the interview and LB recorded and transcribed all interviews verbatim for analysis.

### Data processing and analysis

A qualitative content analysis method developed by Knafl et al. was used for the interpretation, analysis, and summary of cognitive interview data (25). The Knafl et al. analytical process includes the transcription of interview data, a summary of patients’ interpretations of items, a summary of patient-identified problems, a breakdown of item summaries, and modifications to items.

Data were analyzed after each interview, and items of the questionnaires were immediately modified following each interview (22). Interviews continued until we reached saturation. The data saturation point in this study was defined as the point at which no new results were obtained from three consecutive interviews, that is, as long as no new descriptive or conceptual findings are observed in the interviews in three consecutive interviews (18). LB and FA transcribed the data to a Microsoft Word document and used the Dedoose software to collect and analyze the data using a case-by-case approach (i.e., data were collected question-by-question) (26;27). Patient-highlighted issues were identified and categorized into the following pre-determined codes: clarity/understanding, relevance, inadequate response definition, reference point, and perspective modifiers (28). Additional codes that emerged during analyses, were verified by another member of the research team to ensure accuracy. LB and FA independently and in duplicate categorized the data in code and resolved the conflict by discussion or with a third party on the research team. The final version of the CoPaQ was distributed to participants for final feedback.

## RESULTS

### Participants

We invited fifteen eligible participants into the study and recruited all invited fifteen participants who (i) have a history of LBP (*n* = 5, 40 percent female, median age of 60 years (interquartile range (IQR): 26–71.5), median duration of LBP 7 years (IQR: 4–32.5), occupations: retired (*n* = 3), student (*n* = 1), insurance (*n* = 1)); (ii) are researchers (*n* = 5, 20 percent female, median age of 33 years (IQR: 29–45)); and (iii) are primary care professionals (chiropractors and physiotherapists) with expertise in treating LBP (*n* = 5, 80 percent female, median age of 40 years (IQR: 37.5–65)). No primary caregivers were enrolled in this study. Table 1 illustrates the detailed demographic information of individual participants.

**Table 1.** Individual participant demographic information

	Age	Gender (identity)	Level of education	Ethnicity	Duration of LBP	Occupation
*Patients*						
P1	70	Female	Post-Secondary	Canadian	20 yr (on and off)	Retired (Former: Communications)
P2	60	Male	Post-Secondary	British, Canadian	History of LBP 45 yr ago	Retired (former: Police Officer)
P3	33	Male	Post-Secondary	Caribbean	7 yr (on and off)	Insurance Advisor
P4	73	Female	Post-Secondary	Scottish/Canadian	2 yr prior to surgery	Retired (Former: Admin Assistant)
P5	19	Male	Post-Secondary (ongoing)	Arab	6 yr	Student
	Age	Gender (identity)	Level of education	Ethnicity	Profession	Work experience (years)
*Clinicians*						
C1	56	Male	Graduate	Canadian	Physiotherapist	26
C2	30	Male	Graduate	Caucasian	Physiotherapist	7
C3	33	Male	Graduate	Canadian	Chiropractor	4
C4	34	Female	Graduate	Black	Chiropractor	3
C5	28	Male	Graduate	Indo-Caribbean, mix of White/African	Chiropractor	2
*Researchers*						
R1	38	Female	Graduate	Canadian	Researcher	3
R2	37	Female	Graduate	Asian/Latin	Researcher	4
R3	65	Male	Graduate	Canadian	Researcher	42
R4	65	Female	Graduate	Canadian	Researcher	40
R5	40	Female	Graduate	Canadian	Researcher	2

## Themes identified

Our results suggest improvements were needed to improve CoPaQ items. Overall, the content analysis of the transcribed interviews revealed significant sources of error that were categorized into the following themes: (i) organization; (ii) clarity/comprehension; (iii) perspective modifier; (iv) reference point; (v) relevance; (vi) inadequate response definition. Proposed revisions for the CoPaQ for use in LBP populations, pending additional validation testing, can be found in Appendix B of the Supplementary Material.

### Organization

Organization refers to the questionnaire’s structure, flow, and consistency. The results suggest changes to the question order, language, and grouping of similar questions.

Participants recommended improving consistency in word choice, replacing general statements with specific ones (e.g., “LBP” instead of “health condition”), and adding a timeframe reminder. The questionnaire lacked skip logic or had it placed incorrectly, causing respondents to not be able to answer applicable questions. Participants also suggested reorganizing the questions in chronological order, and group questions based on similar themes to improve flow and reduce overlap in different sections.

- C3: “I’m not sure if this [question] fits this category of your questionnaire. Because I believe the next section has to do more with time. And it might be better suited in that section.”

The proposed modifications to the organization include grouping related questions, adjusting the skip logic, and only asking relevant questions to the respondents.

### Clarity/comprehension

Clarity was achieved by defining unclear or ambiguous areas of the questionnaire. This involved rephrasing and simplifying questions to reduce misinterpretation, tailoring the content to the population’s literacy level, and incorporating prompts specific to a population with LBP. The proposed modifications to the questionnaire include adding LBP-specific examples, clarifying sentences, and using concise language for general questions.

### Reference point

The reference point is described as when participants have difficulty responding to a question due to a changed reference point, unclear boundaries, or information that is challenging to recall. Many participants noted it would be challenging to recall information based on the timeframe specified and suggested that the boundaries of some questions be better defined. Questions regarding distance traveled raised recall issues, it was suggested to collect time instead of kilometers for improved accuracy. However, this may present challenges in calculating cost-effectiveness as it is easier to allocate a cost to kilometers (e.g., millage and gas costs) as opposed to time spent traveling.

Defining boundaries within a question was a concern and participants struggled with determining the appropriate level of breadth or narrowness in their interpretation.

- C1: “Do you think you need to define children or not… people could have adult children living with them.”

Proposed modifications to the questionnaire include defining clear boundaries for the question stem to better guide respondents.

### Perspective modifier

A perspective modifier describes how individual life experiences and personal/environmental factors affect a person’s response to a questionnaire item. This theme was primarily identified by the researchers and raised concerns about the definitions used to describe the scales, emphasizing the need for clearly defined anchors, cultural adaptation to account for language and cultural differences, and adjustments for variations in interpretation related to the severity of the respondent’s LBP.

Concerns were specifically raised for Questions 4.1 and 4.2 assessing financial stress. One participant stated:

- R3: “[Y]ou may want to define financial stress. […] I may have an assumption of what it is, but someone else may have a different one. So, in order to contextualize your responses, you may want to by [defining] financial stress.”

Another participant noted that indirect costs may vary depending on the severity of their LBP.

- C2: “I think sometimes people stop education because of—particularly back pain, that [is] either severe [or an] acute bout from some type of injury, or even if it gets bad and fairly chronic like it might stop an undergrad degree or something, and that would be a lost cost for them. Definitely seen that happen before so, that would be something to consider adding.”

### Relevance

This theme was coded when participants thought certain questions seemed irrelevant when considering the purpose of the questionnaire. For example, participants noted costs of additional non-medical services (Q1.14) were already captured by the first question (Q1.1) asking for all care services (including non-medical services) received. Proposed modifications to the questionnaire include removing repetitive questions and combining overlapping/similar questions.

### Inadequate response definition

This theme was coded when there were issues with the connection between questions and responses and missing response options. Suggestions included adding more response items to improve clarity, and options where the participant may not be able to recall the answer (e.g., I do not know, I cannot recall). Participants additionally felt the CoPaQ lacked comprehensive options for some questions, noting potentially missing options in the responses.

- P2: “Okay, so we can actually do them like all that apply here since it might not just necessarily be one [answer] on that.”

### Demographic section

All participants provided feedback for the demographic section. Many participants suggested additional response options for gender identity. Participants suggested adjusting the questions about weight and height to account for different units of measurement (e.g., pounds versus kilograms) and clarifying the question about the use of painkillers by including examples. They also noted missing and unclear options for the question about employment status.

### Verbal probing

To further elicit participants’ thoughts on the questionnaire, the interviewer asked participants for their overall thoughts on the questionnaire and if they felt any elements were missing or problematic. Participants found the questions relevant but suggested modifications for clarity and organization. Many felt the questionnaire was long, yet exhaustive, indicating the CoPaQ effectively captured all necessary aspects of the participants’ experiences of LBP related to direct or indirect costs, ensuring content validity. Participants further suggested adding items for caregivers and indirect costs (e.g., educational loss, loss of income from spouse). One participant mentioned the sensitive nature of the questionnaire and suggested questionnaire administers be mindful of respondents:

- P5: “I think to some people that it might be sort of overwhelming to give to someone because, of course, like if someone has …. financial stress with their health in general, this questionnaire will just force them to like to address it right. […]. I think really just as a practitioner, taking that sort of intuition to know how your patient is feeling in general.”

### Additional feedback

The research team modified the questionnaire with the included feedback and forwarded it to participants for further input. Four of the fifteen participants responded, with one finding their concerns addressed; one not having feedback; one stating they did not have time to provide feedback; and one suggested we define terms like “caregiver” and “LBP” and adjust the phrasing of certain questions for consistency and clarity. Overall feedback and revisions can be found in Table 2.

**Table 2.** Additional participant feedback

Organization	
Participant	Quote
C3	Thinking back with the other question that was talking about … the distance and it was being calculated as from home to the medical center. For consistency, you might want to keep it, essentially, similar… so either the distance to and from the health center or … from home to the health center.
R1	[I]t’s a long questionnaire … you have more chance of people skipping question[s].
P3	If there is a way that you can put both of [the questions] together…
C1	[S]hould you have a question that precedes this one? You know ”Were you employed”… And [the responses] “yes or no,” and then, “if no” then they just skip all of those questions because they are not going to be applicable.
R3	I went from answering your first question 1.1 and it kicks me to 1.8. And so now, even though I may have had visits to the [chiropractor] because I saw them in my facility—so I did not actually travel there, you miss all that information, right?
R4	First, how long did it take you to actually get the appointment, the second one, how long did you travel, and third one, is how long did you wait while you were there? How long did it take you to get home?
Clarity/comprehension	
C1	[T]he items you have listed, they are all medical devices, but I would say they are probably … the most infrequently associated with low back pain.
P3	I would maybe … give them more examples as to what like a homecare service would be.
R5	The other thing that I wanted to point out is that a lot of people probably do not know what net means or do not understand what you mean when you say that.
P5	I feel like that would be a bit hard to read…
Reference Point	
R3	Now, when you say, “are you taking painkillers for your low back pain?” This is a, an open-ended painkiller for over the counter or prescription … [if I] take Advil to, you know, I take an opioid, that is completely different.
Perspective modifier	*No other additional feedback*
Relevance	
R3	So time spent by your caregiver who accompanies you, not directly related to health care services, but now you are asking if they are traveling to the health facility, yeah, does that make sense? If it is not related to their health services, why would they be traveling to the center?
P5	So, I feel like “sometimes” [is] a little redundant, because the question does ask like “did you ever pay for parking?” So, if you ever paid for it then it is like either yes or no
Inadequate response definition	
R4	I mean the other item that looks like it is missing for me, you have lost your job or been laid off because you cannot work, which happens to a lot of people, job loss or layoff.

## Discussion

This cross-sectional qualitative study has demonstrated content validity of the CoPaQ by accurately representing the construct we aim to measure and establishing its relevance, comprehensibility, and comprehensiveness for use in LBP contexts. The content analysis of the interviews revealed significant sources of error that were categorized into the following themes: (i) organization; (ii) clarity/comprehension; (iii) perspective modifier; (iv) reference point; (v) relevance; and (vi) inadequate response definition. Our results suggest improvements to the comprehension and relevance of items in the questionnaire and restructuring the questionnaire to address concerns about organization.

Costs related to care for dependents and whether the respondent was a caregiver themselves were identified. Costs may vary depending on the severity of the LBP and the need for a caregiver. To avoid misinterpretations, revisions were made to clarify boundaries and provide relevant examples in the question. This helps narrow the scope of the question, ensures costs collected are directly related to LBP, and helps prevent overestimation/underestimation of costs. For example, a prompt asking about medication can mislead patients to include unrelated medications, leading to higher reported costs for LBP treatment. Concerns regarding the definition of financial stress and the use of time rather than distance were not addressed, as the goal was to capture a subjective perception of stress and indirect costs (i.e., fuel expenses). Feedback further suggested revisions to the organization. To improve the flow of the questionnaire, we included skip logic to avoid irrelevance for certain subgroups, revised the language for better word choice and consistency throughout the questionnaire, and changed the structure of the questionnaire to help decrease the length of the questionnaire.

Feedback from participants called for consideration of participant’s individualities and emotions when administering the questionnaire. The questionnaire addresses topics that may be challenging to discuss such as financial stressors, interrupted careers or education, caregiving strain, and so forth. Highlighted by a participant in the persons with LBP group, these questions can impact the completion or fullness of responses and adversely impact the participant’s engagement and mood. Researchers should place special consideration on how to administer this questionnaire to ensure accuracy and promote comfort.

Cost diaries are commonly used to assess LBP costs but have limitations such as inconsistent tracking and incomplete cost capture (29;30). The CoPaQ is an alternative method that overcomes these limitations by using a questionnaire to measure both indirect and direct costs but may raise concerns about recall error and length of the questionnaire. Selecting an optimal recall time requires questionnaire administrators to balance the issues of under and overreporting by changing the timeframe in the modified CoPaQ. A study was conducted to assess the accuracy of self-reported disability, comparing self-reported sick days to administrative records over 1-month and 3-month intervals (31). No significant difference in recall accuracy for missed workdays were found, suggesting individuals’ self-reports of missed workdays were as accurate for a 3-month recall as for a 1-month recall. This is consistent with literature assessing recall accuracy at 3-month, 6-month, and 12-month time intervals, suggesting no clear difference in recall accuracy at the different timepoints (29;32;33).

Our study had strengths such as two researchers completing cognitive interviews and analyses for quality assurance and providing a comprehensive investigation of issues in the questionnaire with diverse and qualified participants providing exhaustive descriptions. Data were collected on ethnic origin, education, and employment status to capture potential health disparities. Limitations are that concerns of recall error or questionnaire length cannot be adequately addressed without compromising comprehensiveness. Additionally, there was imbalanced gender in specific groups (R: 20 percent female; C: 80 percent female), and a lack of perspectives from participants with severe LBP and primary caregivers for persons with LBP, which may limit understanding of experiences and perceptions of those who incur additional costs. Additionally, we used a data saturation method for sampling, which, while viewed as vital for sampling and enhancing the quality of qualitative research, introduces subjectivity at the researcher’s discretion to define a saturation point (29). Our study further had a low response rate at the follow-up for additional feedback on the questionnaire, however, those who did respond offered minor to no revisions. Future studies should compare the accuracy of the CoPaQ and cost diaries using LBP cost data as well as pilot studies among groups with higher severity of LBP and collect information on socioeconomic status to identify additional missed perspectives.

## Conclusion

Our study evaluated the content validity of a questionnaire that assesses the direct and indirect costs associated with LBP. This tool is an effective means to gauge an average estimate of costs associated with LBP retrospectively; however, for prospective studies, patient diaries may be more accurate in capturing cost. Our findings suggest improvements to the comprehension and relevance of items in the questionnaire and restructuring the questionnaire to address concerns about organization. Based on the feedback, we have modified the CoPaQ for use among this patient population. Future studies should administer this questionnaire to populations with balanced genders, groups with varying severities of LBP, and perspectives of primary caregivers of LBP, while also considering alternative forms of sampling and different recall periods. Future research should explore the potential benefits of combining quantitative assessment and concurrent validity. Pilot studies consisting of patients with varying levels of LBP severity should be conducted to determine if the length of the questionnaire is an issue and to enhance the robustness of this questionnaire.

## Supporting information

Bakaa et al. supplementary material Bakaa et al. supplementary material

## Data Availability

All data are available in the appendix of the Supplementary Material.

## Supplementary material

The supplementary material for this article can be found at http://doi.org/10.1017/S0266462324000515.
