# Supplementary material for: Content validation of the COST for patient questionnaire (COPAQ) for patients with low back pain: a qualitative study
Source: Int J Technol Assess Health Care. 2024 Nov 5;40(1):e46. doi: 10.1017/S0266462324000515 (PMC11563178; doi:10.1017/S0266462324000515)
Supplement: Bakaa et al. supplementary material [file S0266462324000515sup001.docx]

# Appendix A: Interview Guide

The main purpose of this questionnaire is to assess the costs related to living with low back pain. Our goal, in collaboration with you, is to improve the questionnaire to identify all the costs (direct and indirect) of patients with low back pain.

We now ask you to help us improve this questionnaire by reading and thinking about every question based on your experience.

You should read the questions of this questionnaire carefully, compare it with your cost conditions for LBP disease, and express your opinions and suggestions. Note that at the end of reading this questionnaire, answer these questions orally:

- This questionnaire is meant to measure the costs from the perspective of the patient. Are there elements of costs that you think are missing?

- Although not all cost elements may apply to you personally, do you think that some are irrelevant to people with LBP in general?

- Are there any questions that you thought were difficult to understand? If so, which ones? How could we improve them to make them easier to understand?

- Would you be able to answer each of question? If not, which question seem problematic and why?

- What is your overall thought on this questionnaire?

All your information, comments and suggestions will be kept confidential.

Thank you very much for your time.

**Appendix B. Modified Questionnaire**

LBP - Costs for Patients Questionnaire (CoPaQ)

The purpose of this questionnaire is to assess your expenses as a result of your low back pain. It is divided into three sections and should take you 10 to 15 minutes to complete. Some of the items may not apply to your situation. When answering, please only consider the period ranging from _______ to ______. All your information will remain confidential.

All listed costs or expenses must be associated with your low back pain as it relates to your daily life or be directly linked with the use of health care services needed to treat your low back pain for the period ranging from _______ to ______. For some of the questions, you many have more than one answer.

**Section A – Costs for Patients**

1. Do you have insurance coverage to treat your low back pain?

___Yes ___ No

**If YES,**

Please select the type of coverage (select all that apply):

___ Public ___ Private

**Section 1:** *Costs you need to cover (i.e., the amount you have to pay)*

1. On average, how often have you attended the following health services for your low back pain?

|  | Number of visits |
| --- | --- |
| Personal general practitioner or another general practitioner |  |
| Nurse practitioner |  |
| Physiotherapist |  |
| Chiropractor |  |
| Doctor/nurse in the Emergency department |  |
| Outpatient specialist (consultant, neurologist, orthopedic surgeon, spine surgeon) |  |
| Massage therapist |  |
| Physiatrist |  |
| Osteopath |  |
| Acupuncturist |  |
| Naturopath |  |
| Psychologist/Counsellor |  |
| Other (Please specify): |  |
| Other (Please specify): |  |

1. Did you travel to the [health center selected from Q1.1] to receive an appointment for your low back pain (e.g., hospital, family doctor or other health providers, family medicine group, physiotherapy clinic, chiropractic clinic)?
   ____Yes ____No

If **NO**, please go to **question 1.7**

1. What means of transportation did you use to get to your appointment? **Please select ALL that apply**
   ___Public transit (bus, metro/subway)
   ___Taxi/Ride Share (Lyft, Uber)

___Flight
___Your personal vehicle

___Personal vehicle of the person who went with you
___Other means of transportation (walking, by bicycle)

1. On average, how **much time** and **how many kilometers** did you travel from your place of residence to [health center selected from Q1.1] appointment per visit?

   ____ hour(s) ______ minutes **per visit**

____ kilometer(s) per visit

1. Did you ever pay for parking during your appointment(s)?
   ____Yes ____No

If **YES,**

Please provide the average amount paid per visit for your parking needs: $______ CAD per visit

1. When travelling to [health center selected from Q1.1], did you pay for accommodation (e.g., hotel, Airbnb)?

____Yes ____No
**If YES,**

Please provide the total number of visits requiring accommodation and the average amount paid per night for accommodation:
_____ visit(s) requiring accommodation
$____ CAD per visit

1. Did you ever pay any portion “out of pocket” for your prescribed medication to treat your low back pain that was not reimbursed?

____Yes ____No

**If YES,**

Please provide the average amount paid: $_______

1. Did you pay for over the counter medication or dietary supplements to treat your low back pain (e.g., Aspirin, Voltaren, Tylenol, natural products)?

____Yes ____No
**If YES,**

Please provide the average amount paid: $_______

1. Did you pay for any tests or examinations performed during or following **any** of your appointments related to your low back pain (e.g., blood tests, X-rays, MRI, CT scan)?

____Yes ____No
**If YES,**

Please provide the average amount paid: $_______

1. Did you pay for any additional non-medical services during or following your appointments for your low back pain(e.g., printing/mailing insurance forms, sending photocopies, doctor’s certificate, etc.)?

____Yes ____No
**If YES,**

Please provide the average amount paid: $_______

1. Did you incur expenses for the purchase of any health care equipment to manage your low back pain (e.g., blood pressure monitor, blood glucose monitor, walker, wheelchair, protective underwear, back brace, heat/cold press, exercise equipment (gym mat, therapy ball, rubber bands), etc.)?

____Yes ____No

**If YES,**

Please provide the average amount paid: $_______

1. Did you incur any expenses for home care services because of your low back pain (e.g., rehabilitation, massage therapy, home care services (attendant care, support worker, household chores), etc.)?

____Yes ____No
**If YES,**

Please provide the average amount paid: $_______

1. Did you renovate your home to better accommodate your low back pain (e.g., access for wheelchair, shower rails, raised toilet seat, etc.)?

____Yes ____No
**If YES,**

Please provide the average amount paid: $______

1. Did you incur any other expenses (e.g., cleaning services, meal preparations, etc.)?

____Yes ____No
**If YES,**

Please provide the average amount paid: $____

1. Are you a primary caregiver for any dependent individuals (e.g., children, dependent adults, grandparents, pets, etc.)

____Yes ____No ___Prefer not to answer

If **NO,** please go to **Section 2**

1. Are there any children (under 18 years old) in your household?

____Yes ____No ___Prefer not to answer

**If YES,**

The age of the youngest child living in the household is:_________ months/years

1. Did you pay for someone (e.g., personal support workers, home-caregivers, etc.) to care for your dependents during at least one of your appointments or on a full-time or part-time basis (e.g., childcare, adult care, or pet care)?

____Yes ____No
**If YES,**

Please provide the average amount paid: $____

**Section 2:** *Average time spent (or required) to access appointment*

- 1. Approximately how much time did you spend to book an appointment with the [health center(s) selected in Q1.1] (i.e., over the phone, or to schedule an appointment at the clinic prior to your consultation)?
     _______hour(s) ______minute(s)
  2. On average, how long was your waiting time at the [health center(s) selected in Q1.1] for your appointment?
      _____hour(s) ____ minute(s) ____ Not applicable

**Section 3:** *Costs related to your job because of your low back pain*

1. Have you suffered a loss of income because of your low back pain?
   ____Yes ____No
   If **NO**, please go **question 4.1**

If **YES**, for what reasons (list all that apply to you)
____ Short- or long-term decrease in salary as a result of missing work
____ As a result of receiving a claim for employment insurance
____ Reduced working hours per week (e.g., working 4 days/week)
____ Limited career advancement or salary increase (e.g., cannot request or accept a

promotion

____ Decrease in performance due to low back pain (e.g., commission, sales)

____ Job loss or layoff
____ Other (specify): __________________________

1. What is your rough estimate of the incurred loss of income?

$__________ ____ Difficult to evaluate ____ Prefer not to answer

**Section 4:** *Financial stress caused by your low back pain from _______ to ______?*

1. I feel financially stressed due to my low back pain

___ Not at all
___ A little bit
___ Somewhat
___ Quite a bit
___ Very much

**Section B: Costs for Caregivers**

**NOTE:** This entire section will only be prompted if respondents select YES to having a caregiver.
 **Section 5:** *Costs for the caregiver (i.e., the person who regularly devotes time to help you with your daily activities) or the person who accompanies you, if any*

1. Did a caregiver or anyone else accompany you to your appointment because of your low back pain?

____Yes ____No
If **NO,** please go to **Section C**

1. Did the person accompanying you have to travel to accompany you at your appointment?

____Yes ____No
If **NO,** please go to **question 5.8**

1. Did the person accompanying you travel together with you to your appointment?

____Yes ____No
If **YES,** please go to **question 5.8**
If **NO**,

Please specify the means of transportation used by the person accompanying you
___ Public transit (bus/metro/subway)
___ Taxi/Ride Share (Lyft, Uber)

___ Flight
___ Personal vehicle

___ Other means of transportation (walking, by bicycle)

___ I don’t know

1. On average, how much time and how many kilometers did this person travel from their residence to [health center selected from Q1.1] per visit?
   ____ hour(s) ______ minute(s) **per visit** ____ I don’t know

_______kilometer(s) **per visit** ____ I don’t know

1. Did this person pay for parking at your appointment?

____Yes ____No ____ I don’t know
If **YES**,

Please provide the average amount paid per visit

$____ CAD per visit ____ I don’t know

1. Did the caregiver or the person accompanying you pay for any accommodations while accompanying you to the appointment (e.g., hotel, Airbnb)?

____Yes ____No ____ I don’t know

If **YES,**

Please provide the total number of visits requiring accommodation and the average amount paid per night for accommodation:
_____ visit(s) requiring accommodation ____ I don’t know
$____ CAD per visit ____ I don’t know

1. Did the caregiver or the person accompanying require any training to specifically assist you?

____Yes ____No ____ I don’t know
If **YES,**

Please provide the average amount you paid per visit and the overall duration of the caregivers training

$____CAD per visit ____ I don’t know

_____ hour(s) _____ minute(s) training ____ I don’t know

1. Did your caregiver or the person accompanying you incur any other expenses while accompanying you (e.g., job loss, requesting time off from work)?
   ____Yes ____No ____ I don’t know

If **YES**,

Please provide the average amount paid per visit:

$____CAD per visit ____ I don’t know

**Section 6:** *Time spent by your caregiver or the person who accompanies you*

1. What is the estimated average time per week your caregiver or the person accompanying you spends performing various tasks (e.g., housework, home care, errands)?
   _____ hour(s) _____ minutes per week ____ I don’t know

**Section C – Sociodemographic and Health Questions**

1. Date of form completion (day/month/years): _________________________________
2. Duration of low back pain (in weeks): ___________________________________
3. Gender (identity):

❑ Trans Woman

❑ Trans Man
❑ Indigenous or other cultural

gender minority identity (e.g., two-spirit)

❑ An identity not listed
(please specify): ______

❑ Prefer not to answer

❑ Woman

❑ Man

❑ Gender-fluid/ Nonbinary

1. Sex (biological): ❑Male ❑Female ❑Other (specify): _____ ❑Prefer not to answer
2. Country of Birth: _________ ❑Prefer not to answer
3. Age (years): ___________ ❑Prefer not to answer
4. Race (Select all that apply):

❑ Arab

❑ Black

❑ White/Caucasian

❑ Chinese

❑ Filipino

❑ Japanese

❑ Korean

❑ Latin American

❑ South Asian (e.g., East Indian, Pakistani, Sri Lankan, etc.)

❑ Southeast Asian (e.g., Vietnamese, Cambodian, Thai, etc.)

- West Asian (e.g., Iranian, Afghan, etc.)
- Mixed race
- Another visible minority group (please specify): __________
- Prefer not to answer

1. Ethnicity (Select all that apply):

❑ African – Central or West (e.g., Ghanaian, Liberian, Nigerian, Senegalese)

❑ African – Northern (e.g., Egyptian, Libyan, Tunisian)

❑ African – Southern or Eastern (e.g., Ethiopian, Kenyan, South African, Ugandan)

❑ African – Other African origin

❑ American

❑ Asian – West, Central or Middle Eastern (e.g., Afghan, Iranian, Iraqi, Israeli, Lebanese)

❑ Asian – South (e.g., Bengali, Punjabi, Sri Lankan, Tamil)

❑ Asian – East or Southeast (e.g., Chinese, Filipino, Japanese, Korean, Vietnamese)

❑ Asian – Other Asian origins

❑ Canadian

❑ Caribbean (e.g., Cuban, Dominican, Jamaican, West Indian)

❑ European – British Isles (e.g., English, Irish, Scottish)

❑ European – French (e.g., Breton, French)

❑ European – Western (e.g., Austrian, Dutch, German)

❑ European – Northern (e.g., Danish, Swedish, Norwegian)

❑ European – Eastern (e.g., Czech, Hungarian, Polish, Ukrainian)

❑ European – Southern (e.g., Croatian, Greek, Italian, Portuguese, Spanish)

❑ European – Other European origins (e.g., Basque, Jewish, Roma, Slavic)

❑ Indigenous (First Nations, Inuit, Métis)

❑ Latin, Central and South American (e.g., Argentinian, Brazilian, Mexican)

❑ Oceana (Australian and New Zealand)

❑ Pacific Islands (e.g., Fijian, Hawaiian, Samoan)

❑ Mixed ethnicity

❑ Another (please specify): _____

❑ Prefer not to answer

| 1. Do you currently smoke (e.g., e-cigarettes, tobacco, marijuana, etc.)? | ❑Yes | ❑No ❑Prefer not to answer |
| --- | --- | --- |
| 1. Are you currently taking prescription pain killers for   your LBP (e.g., opioids)? | ❑Yes | ❑No ❑Prefer not to answer |
| 1. Are you currently working/employed? | ❑Yes | ❑No ❑Prefer not to answer |
| **If NO,** are you unemployed because of your low back   pain? | ❑Yes | ❑No ❑Prefer not to answer |

1. What is your current employment status?

|  |  |  |  |  |  |  |  |  |  |
| --- | --- | --- | --- | --- | --- | --- | --- | --- | --- |
| Full time/ full duties | Full time/ modified duties | Part Time/ full duties | Part time/ modified duties | Seeking employment | Retraining | Unemployed | Retired | Not seeking employment (specify reason): | Prefer not to answer |

If employed, please specify your occupation, and position you held:

Occupation:__________________

Start year of employment: ______

1. What is your marital status?

|  |  |  |  |  |  |  |
| --- | --- | --- | --- | --- | --- | --- |
| Married | Single | Divorced | Widowed | Common  Law | Other (specify): | Prefer not to answer |

1. What is the level of the highest educational qualification you have completed?

|  |  |  |  |  |  |  |  |  |  |  |
| --- | --- | --- | --- | --- | --- | --- | --- | --- | --- | --- |
| Elementary Certificate | High  School | DEP (Professional Diploma) | College | CEGEP (Quebec: College of General and Professional Teaching) | University Certificate | Bachelor’s degree | Master’s Degree | Doctorate (MD, PhD) | Other (specify): | Prefer not to answer |

1. Please specify the number of people living in your household?

❑ I live alone ❑I live with one or more individuals ❑ Prefer not to answer

1. Do you live in a rural, urban or sub-urban area?

❑ Urban ❑ Sub-Urban ❑Rural ❑Prefer not to answer

1. What is the approximate gross annual income of your household? Check the income category that applies to you

__< $10,000 __$40,000 – $49,999 __$80,000 – $89,999 __> $150,000

__$10,000 – $19,999 __$50,000 – $59,999 __$90,000 – $99,999 __Prefer not to answer

__$20,000 – $29,999 __$60,000 – $69,999 __$100,000 – $124,999

__$30,000 – $39,999 __$70,000 – $79,999 __$125,000 – $149,999
